# Commentary: Rehabilitation: a key service, yet highly underused, in the management of young patients with sickle cell disease after stroke in DR of Congo

**DOI:** 10.3389/fstro.2024.1230339

**Published:** 2024-02-19

**Authors:** Oyéné Kossi

**Affiliations:** ^1^Ecole Nationale de Formation des Techniciens Supérieurs en Santé Publique et Surveillance Epidémiologique, National School of Public Health and Epidemiology, University of Parakou, Parakou, Benin; ^2^Unit of Neurology and NeuroRehabilitation, University Hospital of Parakou, Parakou, Benin

**Keywords:** sickle cell disease, prevalence, stroke, rehabilitation, children

## 1 Current situation in Benin

I read the article by Boma et al. (2023) describing the current situation and future solutions in the management of young patients with sickle cell disease (SCD) after stroke in the Democratic Republic (DR) of Congo. This publication inspired in me to comment on this important public health topic that dangerously gnaws at our societies in sub-Saharan Africa. In Benin, recent data estimate a 22.3% prevalence of sickle cell trait (hemoglobin S) and a 10.21% prevalence of hemoglobin C, and 4% of the population would be affected by hemoglobin SS homozygosity and hemoglobin SC double heterozygosity (Rahimy et al., 2009; Zohoun et al., 2020). With an estimated population of 13 million, in Benin, the absolute number of the population concerned with this problem would be estimated to be more than 4 million. Unfortunately, to date, to the best of our knowledge, no study has been published on the prevalence of stroke in people with SCD in Benin. Overall, in addition to the neurological symptoms that people with SCD may experience, there are also hip or knee osteoarthritis that are common, leading to chronic pain, which, in turn, can interfere with many aspects of the patient's life, including education, employment, and psychosocial development.

## 2 Potential solutions

In Benin, as in most sub-Saharan African countries, systematic screening for SCD is not a common practice as most sickle cell–trait carriers often are asymptomatic. This lack of systematic screening increases the rate of high-risk marriages and thus maintains the prevalence of the disease in the population (Alhamdan et al., 2007; Zounon et al., 2015). Therefore, the first challenge in the fight against SCD remains primary prevention through strategies that use systematic screening for SCD in the population and doing this in strict compliance with ethical rules. Second, there is a need to improve health education programs for the population, more efforts are needed for the counseling of couples, and good management in timing screening could decrease high-risk marriages and ultimately reduce the prevalence of the disease. In this perspective, public authorities, as well as religious and traditional authorities, can play important roles (Alhamdan et al., 2007; Rahimy et al., 2009; Zounon et al., 2015; Ezugwu et al., 2019).

In addition to preventive measures, health professionals should also be trained to manage symptomatic cases and the long-term clinical symptoms of SCD, including knee and hip osteoarthritis and stroke sequelae for stroke cases. As mentioned by Boma et al. (2023), in Benin, we also face a problem of scarcity regarding qualified professionals in the field of rehabilitation, in particular those specializing in the rehabilitation of children with neurological conditions. Current guidelines recommend that following a stroke, rehabilitation should start as early as possible when the patient's medical state allows it (Bernhardt et al., 2019). In children, rehabilitation should be especially playful while being intensive. It is therefore expected that the professionals involved in the rehabilitation of children will have specific competence. Currently, ~200 physiotherapists work in Benin (Kossi, 2023), and only a few specialize in neurological rehabilitation (*n* = 14), including five specializing in the rehabilitation of children and young with cerebral palsy. It is therefore important to create more training programs for the specialization of not only physiotherapists but also other rehabilitation disciplines as their numbers are very poor in the country (Bonnechère et al., 2022a). For example, there are no occupational therapists in the country and only eight speech-language therapists, all working in Cotonou, the capital.

Third, I definitively agree with Boma et al. (2023) that mobile technologies and virtual reality represent new promising perspectives for the rehabilitation of young with SCD given the scarcity of rehabilitation professionals and the playful aspects of this means, making them fully suitable for this population (Bonnechère et al., 2022a,b; Rintala et al., 2022).

Fourth, promoting a lifestyle with regular physical exercise in young and adults with SCD would also be a promising way to prevent and reduce the negative effects of the disease, including hip or knee osteoarthritis, chronic pain, and stroke. In particular, walking and swimming are more suitable, more affordable, and, overall, more acceptable in most cultures (Noukpo et al., 2022; Nindorera et al., 2023).

## 3 Call to action

Public health authorities in sub-Saharan African countries need to invest more in stroke prevention and managing of stroke cases by setting up national insurance systems to reduce the cost. In addition, researchers and clinicians in the regions need to develop South–South and North–South partnerships to increase patient access to stroke care, in particular for youth and adolescents with SCD. In Benin, we envision developing a technology-supported rehabilitation center, including affordable technology and mHealth solutions at Parakou University Hospital, in the north of the country. This important project is being implemented in collaboration with Hasselt University (Belgium). In view of the findings in the DR of Congo, we believe that a collaborative effort between Belgium, the DR of Congo, and Benin would have a significant impact on the region regarding the rehabilitation of youth and adult SCD victims of stroke.

## Author contributions

OK designed and wrote the manuscript.
