# Erratum: Commentary: Rehabilitation: a key service, yet highly underused, in the management of young patients with sickle cell disease after stroke in DR of Congo

**DOI:** 10.3389/fstro.2024.1404682

**Published:** 2024-04-18

**Authors:** Frontiers Production Office

**Affiliations:** Frontiers Media SA, Lausanne, Switzerland

**Keywords:** sickle cell disease, prevalence, stroke, rehabilitation, children

Due to an editorial error, this article was published with the wrong article type.

The article type should be **General Commentary** and the title was updated accordingly to “*Commentary: Rehabilitation: a key service, yet highly underused, in the management of young patients with sickle cell disease after stroke in DR of Congo*.”

The publisher apologizes for this mistake. The original article has been updated.

